# Nucleoside Transport and Nucleobase Uptake Null Mutants in *Leishmania mexicana* for the Routine Expression and Characterization of Purine and Pyrimidine Transporters

**DOI:** 10.3390/ijms23158139

**Published:** 2022-07-23

**Authors:** Mustafa M. Aldfer, Tahani A. AlSiari, Hamza A. A. Elati, Manal J. Natto, Ibrahim A. Alfayez, Gustavo D. Campagnaro, Bashiru Sani, Richard J. S. Burchmore, George Diallinas, Harry P. De Koning

**Affiliations:** 1 Institute of Infection, Immunity and Inflammation, College of Medical, Veterinary and Life Sciences, University of Glasgow, Glasgow G12 8QQ, UK; 2421701a@student.gla.ac.uk (M.M.A.); 2414533a@student.gla.ac.uk (T.A.A.); 2218613e@student.gla.ac.uk (H.A.A.E.); manal.natto@glasgow.ac.uk (M.J.N.); ibrahim-fa@hotmail.com (I.A.A.); campagnarogd@gmail.com (G.D.C.); bashmodulus@gmail.com (B.S.); richard.burchmore@glasgow.ac.uk (R.J.S.B.); 2 Department of Biology, National and Kapodistrian University of Athens, Panepistimioupolis, 15784 Athens, Greece; diallina@biol.uoa.gr; 3 Institute of Molecular Biology and Biotechnology, Foundation for Research and Technology, 70013 Heraklion, Greece

**Keywords:** equilibrative nucleoside transporter, nucleobase transport, *Trypanosoma cruzi*, *Trichomonas vaginalis*, FurD uracil transporter, *Aspergillus nidulans*, expression system

## Abstract

The study of transporters is highly challenging, as they cannot be isolated or studied in suspension, requiring a cellular or vesicular system, and, when mediated by more than one carrier, difficult to interpret. Nucleoside analogues are important drug candidates, and all protozoan pathogens express multiple equilibrative nucleoside transporter (ENT) genes. We have therefore developed a system for the routine expression of nucleoside transporters, using CRISPR/cas9 to delete both copies of all three nucleoside transporters from *Leishmania mexicana* (ΔNT1.1/1.2/2 (SUPKO)). SUPKO grew at the same rate as the parental strain and displayed no apparent deficiencies, owing to the cells’ ability to synthesize pyrimidines, and the expression of the LmexNT3 purine nucleobase transporter. Nucleoside transport was barely measurable in SUPKO, but reintroduction of *L. mexicana* NT1.1, NT1.2, and NT2 restored uptake. Thus, SUPKO provides an ideal null background for the expression and characterization of single ENT transporter genes in isolation. Similarly, an LmexNT3-KO strain provides a null background for transport of purine nucleobases and was used for the functional characterization of *T. cruzi* NB2, which was determined to be adenine-specific. A 5-fluorouracil-resistant strain (Lmex5FURes) displayed null transport for uracil and 5FU, and was used to express the *Aspergillus nidulans* uracil transporter FurD.

## 1. Introduction

Nucleoside and/or nucleobase transporters are essential to the growth and proliferation of all protozoan parasites. All parasitic protozoa are deficient in de novo purine biosynthesis and several anaerobic protozoa are also deficient in pyrimidine biosynthesis [1]. Purine and pyrimidine transporters are also important conduits for chemotherapeutic agents, particularly in trypanosomes, and thus also play a role in drug resistance [2,3,4]. A well-studied example is the role of the TbAT1/P2 aminopurine transporter of *T. brucei* in the uptake of melaminophenyl arsenicals and diamidines [5,6,7]. In recent years, purine antimetabolites have been shown to be among the most effective classes of antiprotozoal compounds, with promising activities against *Trypanosoma brucei* [8,9,10,11], *T. congolense* [12], *T. cruzi* [13], *Trichomonas vaginalis* [14], and *Leishmania* spp. [15], among others, placing further emphasis on the pharmacological importance of nucleoside carriers. In *T. brucei*, both P1- and P2-type nucleoside transporters are implicated in the uptake of purine antimetabolites [8,11]. Similarly, in *Leishmania* species, cytotoxic purine antibiotics tubercidin and formycin B are documented to be transported by NT1 and NT2, respectively [16,17], and in *Toxoplasma gondii*, the antimetabolite adenine arabinoside (AraA) is taken up by the AT1 carrier [18].

However, in many pathogenic protozoa and other pathogens, knowledge of purine and pyrimidine nucleoside and nucleobase transporters is almost nonexistent, for a variety of reasons related to the fact that transport function can only be studied in a cellular system, in contrast to most enzymes, which can be purified and studied in appropriate cell-free systems. For instance, some species are not easily available in the required amounts and purity from host cell material, and some transporters are only expressed in some life-cycle stages. Another common complication is the expression of multiple similar transporters in the same cell type, leading to major challenges in assigning observed transport to any specific gene product and in determining true kinetic parameters for any one transporter. The cloning and heterologous expression of single genes is an obvious solution, but consensus around a suitable expression system has not materialized. Early studies of protozoan nucleoside transporter genes universally opted for expression in oocytes of the toad *Xenopus laevis* [16,18,19,20,21], following the practice with human nucleoside transporters [22]. While this has undoubtedly been of great impact to the field, this system has significant disadvantages. For instance, it requires a breeding colony of *X. laevis* from which the oocytes are harvested by surgery; the oocytes need to have the right maturity and the expression requires microinjection of cDNA, making experiments with large numbers of time points or concentrations difficult, and for each experiment this must be repeated. All this is expensive. Moreover, the oocytes themselves express multiple nucleobase and nucleobase transporters, complicating the analysis. An alternative has been expression in the yeast *Saccharomyces cerevisiae* [23,24], which had the clear advantages of easy culture, improved reproducibility, and lower cost. Different *S. cerevisiae* strains, lacking specific transporters, can be used for the heterologous expression of specific transporters. However, well-characterized null-backgrounds for the expression of nucleobase and nucleoside transporters in an easy-to-culture protozoan cell type would be much preferable. We have previously expressed protozoan transporters in *T. brucei* lacking either the TbAT1 or the NT8.1/NT8.2/NT8.3 nucleobase transporter locus [25,26,27,28,29].

Here, we present a full set of *Leishmania mexicana* cell lines collectively deficient in the transport of all the purine and pyrimidine nucleobases and nucleosides. Thus, a single cloning of an unknown transporter gene into the expression vector allows screening for uptake of any of the natural purine and pyrimidine substrates as well as pharmacological analogues in a null background. We propose this as the standard system for the expression of protozoan nucleoside/base transporters. The promastigote cells are easy to culture in any amount, and the expression is highly stable as long as the selection antibiotic is included. We demonstrate the utility of the system by successfully expressing a range of known and unknown transporters in the cells, including the *Aspergillus nidulans* uracil transporter FurD [30], which demonstrates that the expression system has utility beyond protozoan transporters only.

## 2. Results

### 2.1. Construction and Verification of ΔNT1.1/Δ1.2 with a Single Transfection

**Figure 1:**
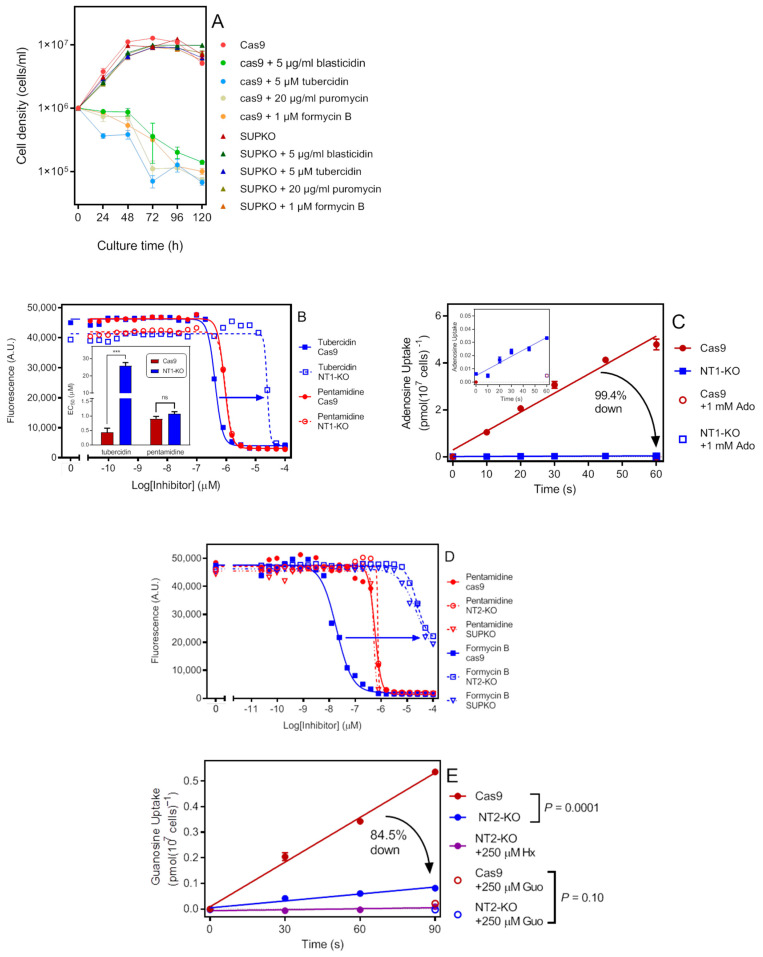
Functional confirmation of NT1 and NT2 knockout in *L. mexicana*. (**A**) Growth curve of cas9 control and SUPKO cells in the presence of selection agents (average of three determinations and SEM). All selection agents led to a progressive decline of cas9 cells but did not impede the growth of SUPKO. (**B**) Resazurin assay with the Cas9 and NT1-KO cell lines. The trace is of a single experiment in duplicate and representative of three identical experiments, the average and SEM of which are depicted in the inset bar chart. *** *p* < 0.001, unpaired *t*-test. (**C**) Transport of 50 nM [^3^H]-adenosine by Cas9 and NT1-KO cells. The rate of uptake was 99.4% lower in NT1-KO (0.00048 ± 0.00007 pmol(10^7^ cells)^−1^s^−1^, r^2^ = 0.93) than in Cas9 cells (0.081 ± 0.006 pmol(10^7^ cells)^−1^s^−1^, r^2^ = 0.98), but both slopes were significantly different from zero (*p* = 0.0021 and *p* = 0.0002, respectively, F-test); the two lines were significantly different *p* < 0.0001. Inset provides a zoom-in of the NT1-KO line. Symbols are the average of triplicate determinations and error bars are SEM; lines were calculated by linear regression. (**D**) Resazurin assay with the Cas9 and NT2-KO cell lines. The trace is of a single experiment in duplicate and representative of three identical experiments, the average and SEM of which are depicted in the inset bar chart. *** *p* < 0.001, unpaired *t*-test. (**E**) Transport of 100 nM [^3^H]-guanosine by cas9 and NT2-KO. The rate of uptake was 84.5% lower in the NT2-KO cells (0.00090 ± 0.00014 (r^2^ = 0.99) vs. 0.0058 ± 0.0003 pmol(10^7^ cells)^−1^s^−1^ (r^2^ = 0.95)). The residual uptake in NT2-KO cells was significantly non-zero (*p* = 0.023) and completely inhibited by 250 µM hypoxanthine or 250 µM guanosine (neither significantly different from zero, *p* > 0.1).

*Leishmania mexicana NT1.1* (LmxM.15.1230) and *NT1.2* (LmxM.15.1240) are situated in tandem on chromosome 15, so a CRISPR/cas9 strategy was devised to delete this locus and replace it with a blasticidin selection marker. In order to select for the deletion of both alleles in one transfection, 5 μM of tubercidin and 5 μg/mL blasticidin were both added to the selection medium, and sensitivity of the cas9 cells to blasticidin and tubercidin at this concentration was confirmed in a growth curve (Figure 1A). Tubercidin is an adenosine analogue and a known substrate of NT1, and has been used previously to select for NT1 mutants [17] and identify the gene [19].

*L. mexicana Cas9/T7 RNAP* cells [31], which constitutively express the humanized *Streptococcus pyogenes* Cas9 nuclease gene and T7 RNA polymerase, were transfected with the sgRNAs (Supplemental Table S1) and the PCR-amplified blasticidin resistance cassette from the pTBlast plasmid for integration into the *NT1.1*/*NT1.2* locus (see Section 4 for details).

The resistance of the NT1-KO to tubercidin was confirmed using a resazurin assay, which showed a 60-fold shift in tubercidin sensitivity (*p* < 0.001 by unpaired *t*-test) whereas the EC_50_ for pentamidine, used as internal control, was unchanged (Figure 1B). Moreover, the rate of uptake of 50 nM [^3^H]-adenosine in the NT1-KO was 99.4% lower than in the parental cas9 cells (Figure 1C).

### 2.2. Construction and Verification of ΔNT2 with a Single Transfection

The *NT2* locus (LmxM.36.1940) was knocked out also in one round of transfection, employing 20 µg/mL puromycin and 1 µM formycin B selection for the selection pressure; sensitivity of the cas9 cells to these concentrations was confirmed with a growth curve (Figure 1A). Details of the gene deletion are given in Section 4. The deletion was confirmed by PCR and qRT-PCR targeting the NT2 open reading frame.

NT2 is an inosine/guanosine transporter and formycin B is a known substrate that has been used before in the cloning and knockout of *Leishmania donovani NT2* [16,32]. The NT2-KO strain was strongly resistant to formycin B compared to the cas9 line (EC_50_ 63.5 ± 1.9 vs. 0.018 ± 0.002 µM, *n* = 3; *p* < 0.0001) (Figure 1D). Uptake of [^3^H]-guanosine was diminished by 84.5% in NT2-KO (*p* = 0.0001), but still significantly different from zero over 90 s (*p* = 0.023). This residual uptake was completely inhibited by 250 µM of either guanosine or hypoxanthine (Figure 1E). This seems to indicate that a hypoxanthine-sensitive transporter, presumably NT3, is able to transport trace amounts of guanosine in the absence of competing nucleobases—a situation similar to the situation in *T. b. brucei* bloodstream forms, where the high-affinity H2 hypoxanthine-guanine displays a moderate affinity for guanosine, but not for inosine [33].

### 2.3. Construction and Verification of Δ(NT1.1/NT1.2/NT2) (SUPKO)

The construction of SUPKO followed the exact protocols outlined above for the construction of ΔNT1 and ΔNT2, but sequentially, meaning that the ΔNT1 cell line was transfected with the guide RNAs, selection cassette, and primers for the deletion of *NT2*, and the ΔNT2 cells transfected with the constructs for the deletion of the *NT1.1*–*NT1.2* locus. Selection was with blasticidin + tubercidin and puromycin + formycin B, respectively, as above. Both approaches yielded viable transfectants, and deletion of all three nucleoside transporter genes was confirmed by PCR and qRT-PCR.

### 2.4. Characterization of Purine and Pyrimidine Transport in SUPKO

**Figure 2:**
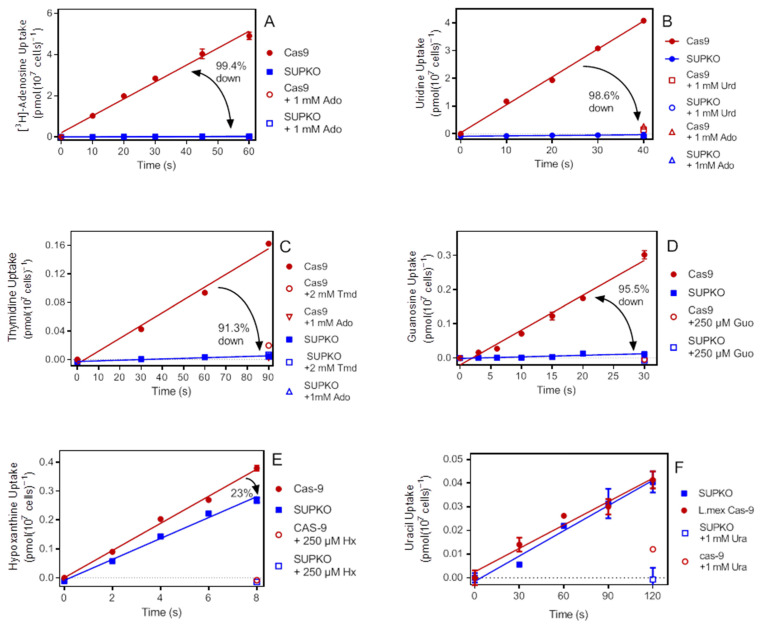
Nucleoside transport in SUPKO compared to Cas9 cells. (**A**) Uptake of 0.1 µM [^3^H]-adenosine was >99% reduced in SUPKO compared to Cas9 (0.00046 ± 0.00002 vs. 0.082 ± 0.004 pmol(10^7^ cells)^−1^s^−1^ (both r^2^ > 0.99; *p* < 0.0001). The uptake in SUPKO was still significantly non-zero over 60 s (*p* < 0.0001). (**B**) 100 nM [^3^H]-uridine was taken up robustly by cas9 cells, but significantly less in SUPKO (*p* < 0.0001; 0.10 ± 0.003 vs. 0.0014 ± 0.00009 pmol(10^7^ cells)^−1^s^−1^ (r^2^ = 0.997 and 0.989, respectively). Uptake in SUPKO was significantly non-zero (*p* = 0.0005). (**C**) Uptake of 50 nM [^3^H]-thymidine was lower in SUPKO than in cas9 cells (*p* = 0.0003; 8.7 × 10^−5^ ± 1.2 × 10^−5^ vs. 0.0018 ± 0.0001 pmol(10^7^ cells)^−1^s^−1^ (r^2^ = 0.962 and 0.988, respectively). The residual thymidine uptake in SUPKO was significantly non-zero (*p* = 0.019). (**D**) Uptake of 0.1 µM [^3^H]-guanosine was different in Cas9 and SUPKO cells (*p* < 0.0001; 0.010 ± 0.0006 vs. 0.00046 ± 0.00011 pmol(10^7^ cells)^−1^s^−1^; r^2^ = 0.98 and 0.78, respectively). Residual guanosine uptake in SUPKO was significantly non-zero (*p* = 0.0085). (**E**) Uptake of 100 µM [^3^H]-hypoxanthine was lower in SUPKO than in cas9 cells. The rates were not significantly different over 3 experiments by unpaired *t*-test, but in the experiment shown, the difference between the two linear regression lines was significant on F-test (GraphPad Prism 9), *p* = 0.0068). (**F**) Uptake of 100 nM [^3^H]-uracil was identical in SUPKO and cas9 cells (0.00032 ± 0.00003 vs. 0.00031 ± 0.00002 pmol(10^7^ cells)^−1^s^−1^, *n* = 3; *p* = 0.81). One representative experiment in triplicate is shown.

The rate of uptake of radiolabeled nucleosides was compared side by side in the SUPKO and cas9 control cell lines. Transport of 0.05 µM [^3^H]-adenosine in cas9 cells over 60 s was linear, with a rate of 0.082 ± 0.004 pmol(10^7^ cells)^−1^s^−1^; this rate was 99% lower in SUPKO (Figure 2A). However, the rate in SUPKO was still significantly non-zero (*p* < 0.0001), the very small amount just measurable and possibly taken up by the LmexNT3 transporter.

Apart from adenosine, *L. mexicana* NT1 transports pyrimidine nucleosides [29]. In three independent experiments, transport of 0.1 µM [^3^H]-uridine was lower in SUPKO than cas9 cells, by 88.3%, 98.6%, and 97.5%, respectively (*p* < 0.0001), but in each case a significant amount of uridine uptake (*p* < 0.05) could just be discerned (Figure 2B). The very small residual uptake was likely mediated by the U1 uracil transporter, as the uridine *K*_i_ for the *L. major* U1 transporter is 10.9 ± 3.2 µM compared to 0.32 ± 0.07 for uracil [34]. Similarly, uptake of 0.1 µM [^3^H]-thymidine was much reduced in SUPKO cells (95.7 ± 1.8%, *n* = 5, *p* < 0.0001), fully inhibited by adenosine, and the residual in SUPKO barely measurable (Figure 2C).

Liu et al. [32] showed that knockout of *LdNT2* resulted in a virtual null transport phenotype for inosine and xanthosine, but not adenosine. Here, we show that uptake of [^3^H]-guanosine was also very strongly inhibited relative to the cas9 cells (95.5%; *p* < 0.0001) (Figure 2D). As an additional control, we measured the uptake of 100 nM [^3^H]-hypoxanthine, which is known not to be a substrate of *L. mexicana* NT1 [29] and is undetectable over background binding in an *L. major* NT3 knockout strain [35]. We surmised that hypoxanthine uptake, mediated by NT3, might be upregulated after the deletion of the nucleoside transporters, but in fact hypoxanthine uptake in SUPKO tended to be somewhat lower than in Cas9 cells (Figure 2E), with an average rate of 0.045 ± 0.004 vs. 0.064 ± 0.007 pmol(10^7^ cells)^−1^s^−1^, *n* = 3), although this did not reach statistical significance (*p* = 0.12, unpaired *t*-test). Although the difference in hypoxanthine uptake was not significant, we followed up with uptake of a pyrimidine base, [^3^H]-uracil, which is taken up by a separate transporter in *L. major* [34] and *in T. brucei* [36] that is presumed not to be encoded by an ENT-family transporter [37]. In three separate experiments, we found that the uptake of 100 nM [^3^H]-uracil in SUPKO or NT1-KO cells was identical to that in cas9 (*p* = 0.81) (Figure 2F).

We conclude that SUPKO is a low-uptake background for purine and pyrimidine nucleosides, but not for purine and pyrimidine bases and should be a sensitive system for the expression of nucleoside transporters.

### 2.5. Re-Expression of Leishmania mexicana NT1.1, NT1.2, and NT2 in SUPKO

**Figure 3:**
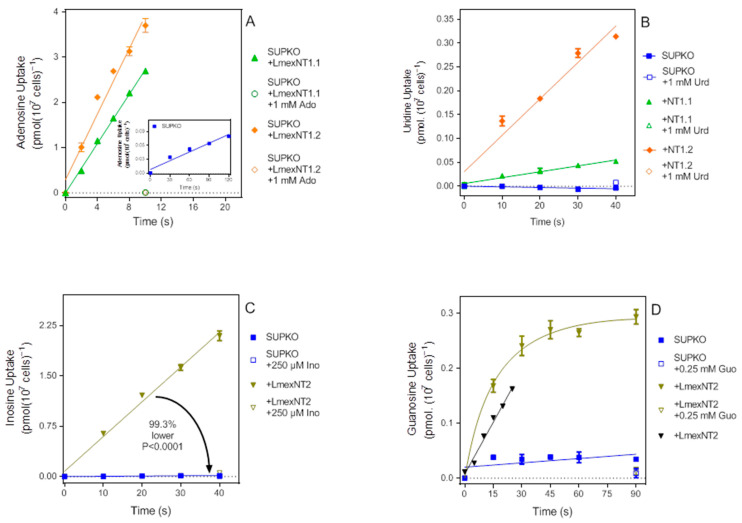
Re-expression of *L. mexicana* nucleoside transporters in SUPKO. (**A**) 50 nM [^3^H]-Adenosine uptake in SUPKO cells expressing LmexNT1.1 or LmexNT1.2. Rates were 0.27 ± 0.006 and 0.36 ± 0.03 pmol(10^7^ cells)^−1^s^−1^, respectively. The two slopes were significantly different (*p* = 0.031; F-test). *Inset:* Uptake in SUPKO was measured over 120 s instead of 10 s because of the lower rate, which was still significantly different from zero (*p* = 0.004) (**B**) Uptake of 50 nM [^3^H]-uridine in SUPKO and the same cells expressing either LmexNT1.1 or LmexNT1.2. The SUPKO rate was not significantly different from zero. Rates for LmexNT1.1 and LmexNT1.2 were 0.0012 ± 0.0001 and 0.0076 ± 0.0009 pmol(10^7^ cells)^−1^s^−1^, respectively, and significantly different from each other (*p* = 0.0004). (**C**) Uptake of 100 nM [^3^H]-inosine by SUPKO and cells expressing LmexNT2. Rates were 0.00039 ± 0.00005 and 0.052 ± 0.002 pmol(10^7^ cells)^−1^s^−1^, respectively, and significantly different (*p* < 0.0001). (**D**) Uptake of 100 nM [^3^H]-guanosine by SUPKO or cells expressing LmexNT2. In the initial experiment, over 90 s, the uptake in the cells expressing LmexNT2 was not linear and the experiment was repeated with the same number of points over just 25 s (black down-triangles). Rates were 0.00039 ± 0.00005 and 0.052 ± 0.002 pmol(10^7^ cells)^−1^s^−1^, respectively, and significantly different (*p* < 0.0001). The rate in SUPKO was not significantly different from zero in this experiment (*p* = 0.24).

In order to test SUPKO as an expression system, we first expressed each of the *L. mexicana* nucleoside transporters separately as “add-back” controls that should restore the respective transport activities. Figure 3A shows linear uptake of SUPKO expressing LmexNT1.1 or LmexNT1.2, with rates of 0.27 ± 0.006 and 0.36 ± 0.03 pmol(10^7^ cells)^−1^s^−1^, respectively (compare 0.00063 for SUPKO (Figure 3A, *inset*). Uridine uptake was also restored in the transfectants, but taken up 6.3-fold faster by cells expressing NT1.2 than NT1.1 (*p* = 0.0004) (Figure 3B). Similarly, expressing NT2 in SUPKO restored the uptake of inosine (Figure 3C) and guanosine (Figure 3D).

### 2.6. Expression of TvagENT3 in SUPKO Equals Expression in T. brucei

**Figure 4:**
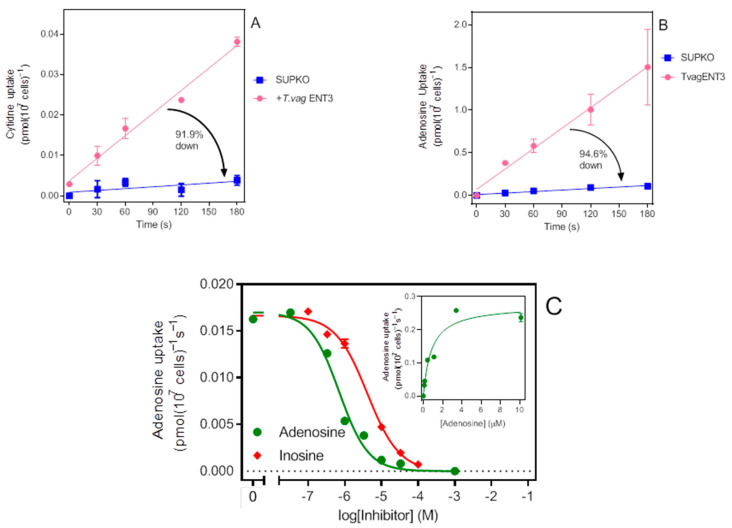
Functional expression of *T. vaginalis* ENT3 in SUPKO. (**A**) 500 nM of [^3^H]-cytidine was significantly higher after expression of TvagENT3 in SUPKO (1.8 × 10^−4^ ± 1.3 × 10^−5^ vs. 1.5 × 10^−5^ ± 9 × 10^−6^ pmol(10^7^ cells)^−1^s^−1^, respectively; *p* < 0.0001). The cytidine uptake in SUPKO was not significantly different from zero (*p* = 0.18). (**B**) Uptake of 50 nM [^3^H]-adenosine was 94.6% lower in SUPKO than in the same cells expressing TvagENT3 (0.00060 ± 0.00007 vs. 0.0080 ± 0.0004 pmol(10^7^ cells)^−1^s^−1^; *p* < 0.0001). Both slopes were significantly different from zero (*p* = 0.004 and 0.0003, respectively). (**C**) Uptake of 50 nM [^3^H]-adenosine in SUPKO over 60 s in the presence of variable concentrations of unlabelled adenosine and inosine. *Inset*: Conversion of adenosine inhibition data to a Michaelis–Menten saturation plot.

As further validation of the expression system, we next tested a known non-kinetoplastid nucleoside transporter, the *Trichomonas vaginalis* transporter TvagENT3, which we recently characterized by heterologous expression in *Trypanosoma brucei* [26]. In that study, TvagENT3 was characterized in the *T. brucei* cell line TbAT1-KO, which lacks the P2/AT1 transporter, but not the array of P1-type nucleoside transporters [38], using [^3^H]-cytidine as the probe. Figure 4A shows that TvagENT3 did strongly increase uptake of 500 nM [^3^H]-cytidine uptake in SUPKO. However, the characterization in *T. brucei* showed that this transporter had the highest affinity for adenosine, followed by inosine, which we were unable to assess in that system because of the expression of multiple P1-type purine nucleoside transporters [39,40,41]. In contrast, the expression in SUPKO showed a very strong induction of [^3^H]-adenosine uptake over background upon expression of TvagENT3 (Figure 4B). The adenosine *K*_m_ value was determined as 0.90 ± 0.13 µM (*n* = 3), identical to the *K*_i_ value found in *T. brucei* (0.87 ± 0.15 µM), and the inosine *K*_i_ as 2.7 ± 0.5 µM (value obtained in *T. brucei* 3.3 ± 0.07 µM; [26]) (Figure 4C). 

### 2.7. Nucleobase and Nucleoside Transport by NT3-KO Cells

**Figure 5:**
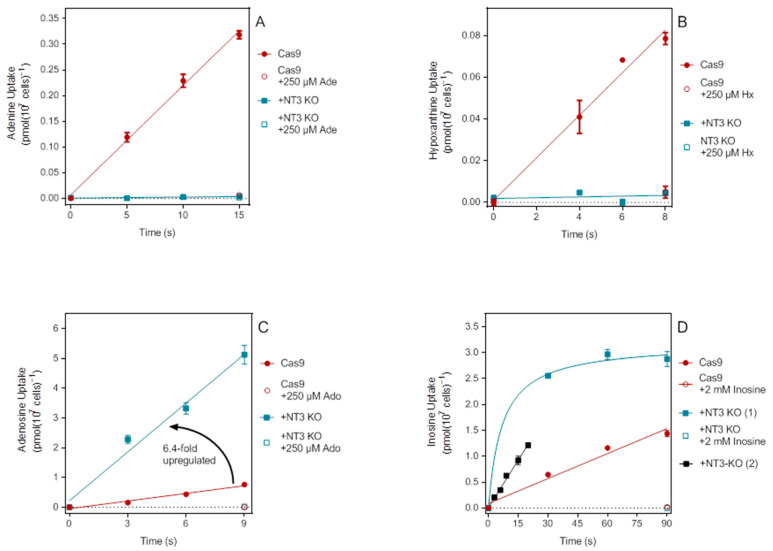
Characterization of purine transport in LmexNT3-KO. (**A**) Transport of 100 nM [^3^H]-adenine by *L. mexicana* cas9 and NT3-KO promastigotes. In NT3-KO cells the rate of adenine transport was <1% of that in cas9 cells (0.000207 ± 0.000094 vs. 0.0213 ± 0.0009 pmol(10^7^ cells)^−1^s^−1^; *p* < 0.0001) and not significantly different from zero (*p* = 0.16), whereas in cas9 cells the slope was significantly different from zero (*p* = 0.0019). (**B**) No significant uptake of 100 nM [^3^H]-hypoxanthine could be detected in NT3-KO cells (*p* = 0.72), but this was highly significant in the cas9 parental cells (*p* = 0.075). The respective rates were 0.00019 ± 0.00045 and 0.0102 ± 0.0009 pmol(10^7^ cells)^−1^s^−1^ (*p* = 0.0006). (**C**) Upregulation of [^3^H]-adenosine uptake (100 nM) in NT3-KO cells compared to cas9 cells. Adenosine uptake in the two cell lines was significantly different (*p* = 0.0010): 0.55 ± 0.05 vs. 0.086 ± 0.009 pmol(10^7^ cells)^−1^s^−1^. Uptake in both strains was significantly different from zero (*p* = 0.0094 and 0.012, respectively). (**D**) Uptake of 100 nM [^3^H]-inosine in cas9 and NT3-KO cells. In a first experiment, transport was measured at 30, 60, and 90 s, which linear for cas9 cells (r^2^ = 0.97; not significantly non-linear by runs test *p* = 0.67; 0.016 ± 0.002 pmol(10^7^ cells)^−1^s^−1^) while the uptake in NT3-KO cells was clearly non-linear over this interval. A reexamination of 100 nM inosine uptake in NT3-KO cells over 20 s showed linearity (r^2^ = 0.99, not significantly nonlinear, *p* > 0.999, and almost 4-fold higher than in the cas9 cells (0.061 ± 0.002 pmol(10^7^ cells)^−1^s^−1^).

An *L. mexicana* knockout strain of NT3 was recently generated using CRISPR technology by Dr Richard Burchmore and his group [42] and was made available to us for characterization with respect to nucleobase and nucleoside transport. A similar NT3-KO cell line was first made in *L. major* by Ortiz et al. [35], after the identification of the gene by the same group [43]. Here, we are primarily concerned with its uses as an expression system for purine nucleobase transporters and as such verified the levels of uptake of a number of purines. Uptake of 100 nM [^3^H]-adenine in NT3-KO was >100-fold lower than in cas9 cells over 15 s and not significantly different from zero (*p* = 0.15) over 15 s (Figure 5A). Uptake of 100 nM [^3^H]-hypoxanthine was particularly fast in cas9 cells and was measured over 8 s (Figure 5B); no significant hypoxanthine uptake was detected in the NT3-KO cells (*p* = 0.72). It thus appears that the NT4 nucleobase transporter, which in *L. major* contributes to adenine uptake at neutral pH but is more important in the intra-macrophage amastigote stage [35], does not play a significant role in nucleobase salvage in *L. mexicana* promastigotes. This probably explains the pronounced growth defect of the *L. mexicana* NT3-KO (see below) also observed for the *L. major* NT3-KO, which was restored to wild-type growth rates upon episomal expression of LmajNT3 [35]. The loss of purine nucleobase transport in the LmexNT3-KO was partially compensated by the upregulation of NT1 and NT2 activity. Figure 5C shows the strong upregulation of 100 nM [^3^H]-adenosine uptake in the NT3-KO. In two separate experiments, the rate increase was 4.0- and 6.4-fold relative to cas9 cells. In separate experiments the uptake rates of [^3^H]-thymidine and [^3^H]-cytidine were also upregulated, by 3.45 and by 3.0- and 3.7-fold, respectively; all these are substrates of the NT1 transporters [29]. Similarly, uptake of the NT2 substrates inosine and guanosine was also upregulated. Figure 5D shows the upregulation of inosine uptake by 4-fold in NT3-KO cells, and guanosine uptake was upregulated by 1.7- and 2.1-fold (both *p* < 0.0001) in two separate experiments. In summary, the uptake of purine nucleobases in LmexNT3-KO cells was not significant over the course of the assay time and, conversely, uptake of nucleosides through NT1 and NT2 was consistently found to be higher than in the cas9 control cells.

### 2.8. Expression of TcrNB2 in NT3-KO

Ortiz et al. [35] have already demonstrated that the episomal expression of NT3 restores wild-type levels of nucleobase transport in *L. major* promastigotes. Instead of repeating this experiment, we chose to introduce the *Trypanosoma cruzi* NB2 transporter into the *L. mexicana* NT3-KO cells instead. We have previously reported the characterization of three of the four *T. cruzi* ENTs by expression in *T. brucei* procyclics, but the substrate for TcrNB2 could not be identified in that expression system [27]. However, phylogenetic analysis showed that TcrNB2 aligned closest to *Leishmania* NT4, which is an adenine transporter at neutral pH [35,44]. Unfortunately, the *T. brucei* NBT-KO strain in which the transporter was expressed retained a high background rate of adenine uptake, although its main locus of three ENT-family nucleobase transporters was deleted [45] and uptake of other purine nucleobases was very low in these cells [27]. The availability of the LmexNT3-KO cell line now allowed us to test our hypothesis. TcrNB2 was cloned into pNUS, and introduced into the NT3-KO and the NT1-KO strains. Clones with high expression were selected by qRT-PCR. 

**Figure 6:**
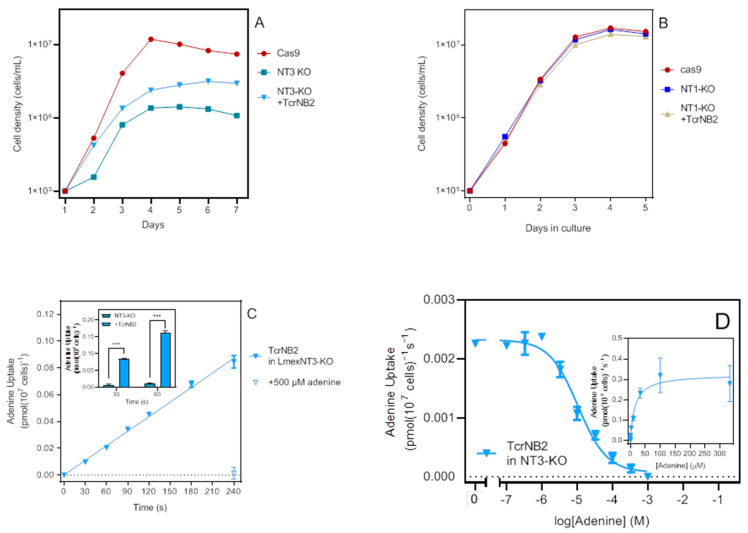
Characterization of TcrNB2 in LmexNT3-KO cells. (**A**) Growth curve of cas9, NT3-KO and NT3-KO expressing TcrNB2. Symbols are the average of triplicate determinations. (**B**) Growth curve of cas9, NT1-KO and NT1-KO expressing TcrNB2. (**C**) Uptake of 100 nM [^3^H]-adenine in NT3-KO expressing TcrNB2 was linear (r^2^ = 0.996; not significantly non-linear, *p* = 0.20) and displayed a rate of 0.00036 ± 0.00001 pmol(10^7^ cells)^−1^s^−1^. *Inset*: separate experiment (*n* = 3) of [^3^H]-adenine uptake by NT3-KO and NT3-KO+TcrNB2 cells. *** *p* < 0.0001 by unpaired *t*-test. (**D**) Uptake of 100 nM [^3^H]-adenine in the presence of various concentrations of unlabelled adenine. Inset: conversion to a Michaelis–Menten saturation plot. The experiment shown is one of three independent repeats, yielding a *K*_m_ of 14.99 µM; incubation time was 120 s.

The expression of TcrNB2 increased the growth rate of the LmexNT3-KO cells, although not to the level of the cas9 parental cell line (Figure 6A), but did not change the growth rate of NT1-KO cells (Figure 6B). Whereas TcrNB2 did not increase the uptake of 50 nM [^3^H]-adenosine, 100 nM [^3^H]-hypoxanthine or 100 nM [^3^H]-uracil when expressed in NT1-KO cells (data not shown), [^3^H]-adenine uptake in NT3-KO cells was readily detectable, linear, and significantly different from zero (*p* < 0.0001) (Figure 6C) as well as highly significantly different from NT3-KO cells (*p* < 0.0001; Figure 6C, inset). An average *K*_m_ value of 14.2 ± 2.6 µM and a V_max_ of 0.26 ± 0.04 pmol(10^7^ cells)^−1^s^−1^ was obtained (*n* = 3) (Figure 6D). Consistent with our observations in the *T. brucei* expression system [27], TcrNB2 appears to be an adenine-only transporter, in that it displayed very low affinity for other purine nucleobases (hypoxanthine) and for adenosine, with 1 mM of these compounds inhibiting adenine uptake by only 31.1 ± 4.3% and 15.5 ± 11.7%, respectively.

### 2.9. Expression of the Aspergillus nidulans FurD Transporter in the L. mexicana Cell Line 5FU-Res

We have previously described the adaptation of *L. mexicana* promastigotes to 5F-uracil and the characterization of the resulting Lmex-5FURes strain, which was almost completely deficient in the uptake of [^3^H]-uracil and [^3^H]-5F-uracil [29]. It was shown that this is due to the loss of the U1 uracil-specific transporter [34], as uridine uptake was unaffected in Lmex-5FURes cells and the cells should therefore be an ideal background for the expression and characterization of (putative) uracil transporters. In order to test this possibility, we subcloned the *Aspergillus nidulans* uracil transporter FurD [30] into the pNUS-HcN plasmid for episomal expression in Lmex-5FURes. Three independent clones were generated and the expression levels were screened using qRT-PCR, the highest-expressing clone being selected. 

**Figure 7:**
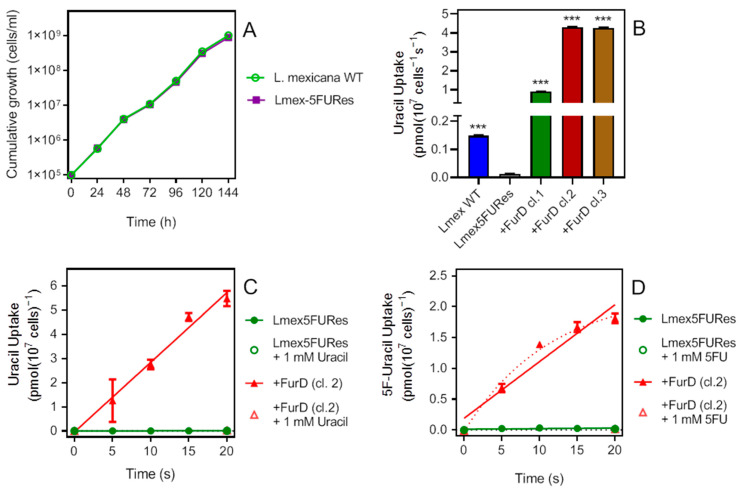

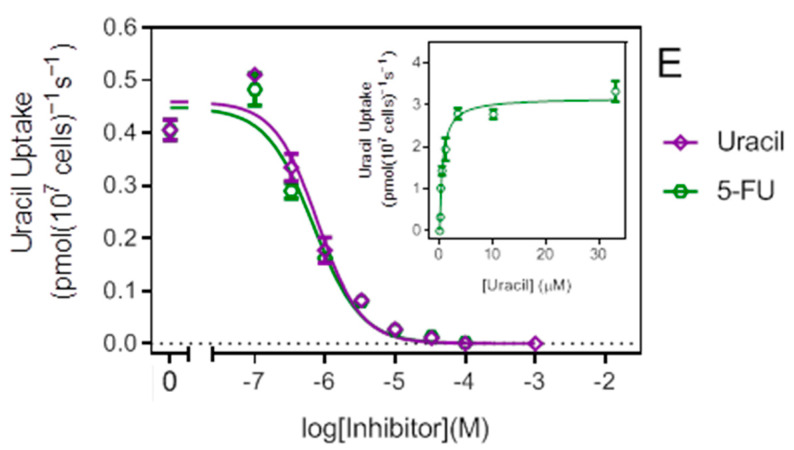
Characterization of *Aspergillus nidulans* FurD in *L. mexicana* 5FURes cells. (**A**) Growth curve of cultures of 5FURes and the parental WT strain. Cells were seeded at a density of 1 × 10^5^ cells/mL, and grown in standard HOMEM supplemented with 10% FCS and 1% penicillin/streptomycin. Cell densities were determined every 24 h. This result represents data from three similar independent repeats. (**B**) Uptake of 100 nM [^3^H]-uracil by WT *L. mexicana* promastigotes and by promastigotes of Lmex5FURes and three clones of that strain expressing *A. nidulans* FurD. Incubation time was 120 s; *** *p* < 0.0001 relative to Lmex5FUres, unpaired *t*-test. (**C**) Uptake of 100 nM [^3^H]-uracil Lmex5FUres and the same cells expressing FurD, clone 2 (cl.2). Rates were 0.00057 ± 0.0001 and 0.29 ± 0.02 pmol(10^7^ cells)^−1^s^−1^ (*p* < 0.0001). (**D**) Like frame C but using 100 nM [^3^H]-5FU as probe. 5-FU uptake was non-linear (dotted line); uptake in Lmex5FURes was not significantly different from zero (*p* = 0.27). (**E**) Uptake of 100 nM [^3^H]-uracil by Lmex5FURes+FurD cl.2 over 10 s. *Inset* is the conversion of the uracil inhibit plot to a Michaelis–Menten saturation plot.

*AnFurD* differs from the other heterologously expressed transporters used in this study, in that it is a member of the NCS1 gene family [46] rather than the ENT transporter family, which, to date, has not been found in any protozoan species [47,48]. Figure 7A shows that promastigotes of the *L. mexicana WT* and the derived 5FURes strain displayed the same growth rate in standard HOMEM medium. The expression of this *Aspergillus* transporter greatly increased uracil uptake in all three clonal strains tested (*p* < 0.0001), very much above even the level in WT *L. mexicana* (Figure 7B). Over 20 s, uracil was very robust in the cells expressing FurD but virtually undetectable in the Lmex5FURes at 0.02% of the rate (Figure 7C). Similarly, uptake of 5FU was rapid in the +FurD cells but not significantly different from zero in the control (Figure 7D). The nonlinearity of the FurD-expressing cells for 5FU uptake can be ascribed to a low rate of metabolic incorporation of 5FU compared to uracil, allowing a buildup of the free substrate in the cell. The *K*_m_ for uracil was determined at 0.89 ± 0.20 µM and the *K*_i_ for 5FU as 0.70 ± 0.25 µM, showing that the transporter does not discriminate by binding affinity between the two substrates (*p* = 0.65) (Figure 7E). This compares with a reported *K*_m_ of 0.45 µM for uracil and *K*_i_ of 0.46 for 5FU in *A. nidulans* [30], showing that *L. mexicana* is an excellent expression system for *Aspergillus* transporters.

## 3. Discussion

In this paper, we report the construction and/or characterization of a series of *L. mexicana* cell lines that display null or extremely low background uptake of either adenosine and pyrimidine nucleosides (NT1-KO), oxopurine nucleosides (NT2-KO), all purine nucleosides (SUPKO), purine nucleobases (NT3-KO), or uracil and 5FU (Lmex5FURes). The construction of the NT1-KO and NT2-KO and SUPKO are reported herein and utilized the CRISPR/cas9 cell lines, tools and vectors developed by Beneke et al. [31]. These were also used to make the NT3-KO cell line, as reported [42], whereas 5FURes was developed through adaptation to gradually increased levels of 5FU in vitro [29]. 

Kinetoplastids are useful systems for the heterologous expression of transporters. For instance, we have previously expressed the *T. congolense* transporter TcoAT1 in the *T. brucei* cell line lacking the TbAT1/P2 transporter [25], showing it did not have the proposed role in diminazene resistance [49], a conclusion recently confirmed by meticulous work on diamidine uptake in *T. congolense* [50]. We have also recently published the first characterization of *Trichomonas vaginalis* nucleoside transporters by expression in *T. brucei* [26] and reported a unique substrate binding mode for *Toxoplasma gondii* oxopurine transporter Tg244440 [28] expressed in a *T. brucei* cell line from which the main nucleobase transporter locus has been knocked out [27]. The characterization of individually expressed transporters is important because it unambiguously links the transport activity to a specific gene product, and disentangles the (potentially) multiple gene products contributing to the uptake of a certain substrate. *T. brucei*, for instance, encodes a dozen ENT transporters [1] as well as additional carriers for uracil [36,51], adenine and hypoxanthine that are apparently not encoded by any of the ENT members [37,45]. Only when it is certain that a single transport function is being measured can a meaningful characterization of its kinetic parameters, substrate specificity and binding mode be determined. 

Beyond the direct insights into parasite biochemistry and physiology that studies of single transporters provide, they have potential pharmacological significance. The study of nucleoside transporters in protozoa was greatly incentivized with the discovery that the TbAT1/P2 transporter of *T. brucei* was responsible for the internalization of essential arsenical [5,52] and diamidine drugs [53,54,55]. It has also long been known that protozoan nucleoside transporters mediate the uptake of cytotoxic nucleoside analogues including formycin B and tubercidin used in this study but also, e.g., adenosine arabinoside in *T. gondii* [18] and cordycepin in *T. brucei* [10,56]. Very recently, a class of 7-substituted,7-deaza analogues of adenosine and inosine have shown great promise against multiple protozoan pathogens [9,12,13,14,57,58], which is clearly mediated by uptake through nucleoside transporters [8,9,10]. 

The *Leishmania mexicana* cell lines we describe here seem highly suitable for the characterization of single transporters. Stable expression of single *L. mexicana* transporters NT1.1, NT1.2. and NT2 will allow the unambiguous characterization of binding motifs and substrate selectivity of each transporter—something that is much harder in the wild-type cells, or even by cloning each gene separately in the more complex background of TbAT1-KO *T. brucei* [29]. Moreover, we expressed *T. cruzi* NB2 in the LmexNT3-KO as phylogenetic analysis had indicated it might be an adenine transporter. We had previously expressed it in the *T. brucei* NB-KO strain but this retains a high background for adenine [27,45]. That study found no evidence that TcrNB2 was able to transport nucleosides or oxopurine nucleobases. Here, we show that TcrNB2 is indeed an adenine transporter, with almost no affinity for adenosine or hypoxanthine. This work also shows that expression of a purine nucleobase transporter in the NT3-KO rescues the significant growth phenotype, and the strain can thus be used to screen for putative nucleobase transporter genes. The confirmation of TcrNB2 as an adenine-specific transporter concludes the characterization of the four *T. cruzi* ENT genes that we identified in its genome: TcrNB1 is a hypoxanthine/guanine carrier, TcrNT1 is an inosine/guanosine transporter and TcrNT2 is a thymidine transporter [27].

Expression of non-kinetoplastid transporters is also possible: *T. vaginalis* ENT3 was successfully expressed in SUPKO and the kinetic parameters matched those of its initial characterization [26]. Further characterization of the nine TvagENTs is now in progress in these cell lines. Finally, we expressed the main uracil transporter from the filamentous fungus *Aspergillus nidulans* in the 5FURes line. This induced very strong transport activity for both uracil and the cytotoxic analogue 5FU, with *K*_m_ and *K*_i_ values very close to those first reported in *A. nidulans* [30]. This leads to the idea that uncharacterized fungal transporters, including proteins of important pathogens (e.g., *A. fumigatus, Candida* species, antibiotic-resistant Mucorales, etc.) can be kinetically studied in specific protozoan cell lines lacking similar endogenous transport activities, as those described herein. From a more fundamental point of view, the nearly identical kinetic characteristics of functionally expressed fungal transporters in protozoa strongly suggest that subcellular tracking mechanisms and specific transporter–membrane lipid interactions [59,60] are functionally conserved between fungi and protozoa, opening a new avenue of cell biology, and biochemical and structural studies of fungal and maybe metazoan transporters in protozoa.

In summary, we report here the characterization of a set of nucleoside and nucleobase transport-deficient *L. mexicana* strains that can be utilized for the functional expression of protozoan and fungal transporters. These tools will be particularly useful for organisms that are not easily cultured or otherwise obtained in large amounts for transporter studies. As such, we have cloned multiple *Trypanosoma vivax* ENT genes for expression in our *L. mexicana* strains. Moreover, some protozoan transporters will only be expressed in life-cycle stages that are not readily available, e.g., *T. gondii* bradyzoites, multiple *Plasmodium* liver and insect stages, *T. brucei* metacyclics or the obligate intracellular amastigotes of trypanosomatids incl. *T. cruzi*, so heterologous expression is certainly the best way for such studies. The characterized transport activities can then be linked back to the life-cycle stages by expression profiles. 

## 4. Materials and Methods

### 4.1. Strains and Cultures

Our wild-type *L. mexicana* strain is MNY/BZ/62/M379, and the 5FURes strain was developed from it by in vitro adaptation to gradually increasing concentrations of 5-fluorouracil (5FU) in vitro [29]. The *L. mexicana-Cas9 T7* strain (derived from *L. mexicana WT* promastigotes by expression of the *Streptococcus pyogenes* Cas9 nuclease gene) is named cas9 in this study and maintained on 32 μg/mL hygromycin [31]. The NT1-KO (ΔNT1.1,ΔNT1.2), NT-2-KO (ΔNT2) and SUPKO strains were made from this using the CRISPR/cas9 protocols described [31], details below. The NT3-KO strain (ΔNT3) was similarly produced from the cas9 strain [42].

All *Leishmania* strains were grown as promastigotes, in standard HOMEM (GIBCO, Life Technologies, Paisley, UK) supplemented with 10% heat-inactivated fetal bovine serum (FBS; PAA Laboratories, Linz, Austria) and 1% of a penicillin–streptomycin solution (Life Technologies) at 25 °C, as described [61]. In order to prepare stabilates of *L. mexicana* promastigotes, cell cultures were added to an equivalent volume of HOMEM containing 30% sterilized glycerol in cryogenic tubes. Before the cryogenic tubes were transferred to liquid nitrogen store, they were frozen at a temperature of −80 °C for 72 h. For the recovery of stabilates, the cryogenic tubes were brought out from the liquid nitrogen store, thawed at 25 °C and then cultivated in HOMEM. The cells were renewed after 20 passages by bringing out fresh cells from the liquid nitrogen store.

### 4.2. Radiochemicals

The following radiolabeled compounds were used during the project: [methyl-^3^H]-thymidine (20 Ci/mmol) and [^3^H(G)]-hypoxanthine (16.1 Ci/mmol) were obtained from PerkinElmer (Waltham, MA, USA). [2,8-^3^H]-adenine (32.2 Ci/mmol) was from NEN Life Science Products (Boston, MA, USA). [8-^3^H]-guanosine (12.9 Ci/mmol) and [6-^3^H]-5-Fluorouracil (20 Ci/mmol) were from Moravek Biochemicals (Brea, CA, USA). [2,8-^3^H]-adenosine (40 Ci/mmol), [5,6-^3^H]-uridine (60 Ci/mmol), [8-^3^H]-inosine (20 Ci/mmol), [5,6-^3^H]-uracil (40 Ci/mmol), and [5-^3^H]-cytidine (20 Ci/mmol) were from American Radiolabeled Chemicals (ARC, St-Louis, MO, USA).

### 4.3. Growth Curves

The growth rates of the *L. mexicana* promastigotes were determined in the standard HOMEM medium supplemented with 10% FBS, in 12-well plates, in triplicate. After every 24 h, cells were counted in a sample of the culture, using either a Neubauer hemocytometer chamber (Hawksley, Birmingham, UK) or by a coulter particle counter and size analyzer (Beckman, Brea, CA, USA) to count the cells in triplicate. An average of the triplicate readings was taken and plotted using GraphPad Prism 8 software to obtain the growth curves.

### 4.4. Molecular Cloning Techniques

The sequences of the nucleotide and amino acid for a gene of interest were obtained from the TritrypDB (tritrypdb.org/tritrypdb; accessed 18 February 2020) website [62]. The sequence alignments and the primers that were used in this project were designed by using the CLC Genomics Workbench version 7.0 software package (CLC bio, Qiagen, Singapore). All the primers used in this project were synthesized by Eurofins MWG Operon (Ebersberg, Germany) and Sigma-Aldrich (Dorset, UK). PCR amplification for sequencing or cloning was performed using Phusion High-Fidelity DNA Polymerase (New England BioLabs, Hitchin, UK), whereas routine screening of gene presence would use GoTaq DNA Polymerase (Promega, Southampton, UK). PCR reaction products were separated on a 1% or 2% of agarose gel with 50 μL/L of SYBR Safe DNA gel stain (Invitrogen) and visualized under UV light.

Expression of genes in *L. mexicana* used the episomal expression vector pNUS-HcN which contains a *G418* resistance gene [63]. These included *T. cruzi* NB2 (tritrypdb TcCLB.506445.110; TcrNB2), *T. vaginalis* ENT3 (TVAG_271560; TvagENT3), *L. mexicana* NT1.1 (tritrypdb LmxM.15.1230), *L. mexicana* NT1.2 (LmxM.15.1240), *L. mexicana* NT2 (LmxM.36.1940) and *A. nidulans* FurD (ENA accession number ABR22526). The appropriate restriction enzymes were used to digest the pNUS-HcN plasmid and the gene of interest (GOI), ligation was performed with T4 DNA Ligase (Promega) or used the NEBBuilder HiFi DNA Assembly Cloning Kit (New England BioLabs), and followed by transfection into *E. coli* XL1-blue cells (Agilent, Stockport, UK) by heat shock, following the manufacturer’s protocol. Colonies were screened by PCR screening using the forward primer of the GOI and the reverse primer of pNUS-HcN plasmid (HDK340). Plasmid from PCR-positive clones were sent to Source Bioscience (Livingston, United Kingdom) for Sanger sequencing. 25 µg of the verified plasmids (pHDK245 ‘FurD’ and pHDK271 ‘TcrNB2′) were precipitated by ethanol and resuspended in 15 µL sterile water.

*L. mexicana* promastigotes (5 × 10^7^ cells) were washed with 100 µL transfection buffer and mixed with 10 µg DNA of the expression construct. The cells were electroporated with the Amaxa Nucleofector (Amaxa AG, Cologne, Germany), using Program U-033. Transfected cells were transferred to 20 mL HOMEM medium containing 10% FBS and allowed to recover overnight at 25 °C after which 50 μg/mL of G418 was added to the culture as a selection agent. To select single cells by limiting dilution the cells were plated out in a 96-well plate to produce individual clones. PCR was used to screen the positive clones, the forward primer of the GOI and the reverse primer of pNUS-HcN plasmid (HDK340) were used to confirm the presence of the target gene in the new strain. The Macherey-Nagel NucleoSpin Tissue kit (Fisher Scientific, Loughborough, UK) was used to extract genomic DNA, according to the instructions given by the manufacturer.

### 4.5. Construction of Knockouts Using CRISPR/cas9

Knockout clones of ΔNT1.1,ΔNT1.2 (NT1-KO) and ΔNT2 (NT2-KO) were prepared using the CRISPR/cas9 system developed by Beneke et al. [31]. The LmexNT1.1/NT1.2 locus was replaced with a blasticidin selection cassette (pTBlast) in the presence of 5 μM tubercidin, which selected for the replacement of both copies of the NT1 locus, as the retention of one allele will allow sufficient tubercidin uptake to be lethal. Similarly, the LmexNT2 allele was replaced with a puromycin (pTPuro)-resistant cassette in the presence of 1 μM formycin B, a cytotoxic inosine analogue taken up by the NT2 transporter. Primers and single guide RNAs (sgRNAs) were designed using the online platform LeishGEdit and are listed in Supplemental Table S1. 

For the PCR reaction for the amplification of 5′ sgRNA and 3′ sgRNA templates (50 µL total volume), PCR steps were 30 s at 98 °C followed by 35 cycles of 10 s at 98 °C, 30 s at 60 °C, and 15 s at 72 °C. This reaction (3 µL) was run on a 2% agarose gel to check for the presence of the expected product. The remainder was heat-sterilized at 94 °C for 5 min and transfected without further purification. The pTBlast and pTPuro plasmids were amplified using primer pairs HDK1742/1743 and HDK1755/1756, respectively, as follows: 110 °C, followed 5 min at 94°C followed by 40 cycles of 10 s at 94 °C, 30 s at 65 °C, 20 s at 72 °C. This reaction (3 µL) was run on a 1% agarose gel to check for the presence of the expected product. The remainder was heat-sterilized at 94 °C for 5 min and transfected without further purification.

For the transfection, 1 × 10^7^ cells of cell line *L. mexicana* Cas9 T7 promastigotes (maintained in the presence of 32 μg/mL of hygromycin) were washed with 150 µL of transfection buffer and mixed with 100 µL of the heat-sterilized mixture of the sgRNA templates and the resistance cassettes in 2 mm electroporation cuvettes. Electroporation was carried out using an Amaxa Nucleofector, program X-001 (Amaxa AG, Cologne, Germany). Immediately, the cells were transferred to 20 mL HOMEM medium with 10% FBS and incubated at 25 °C. The cells were allowed to recover overnight, after which 5 μg/mL of blasticidin and 5 μM of tubercidin (NT-KO) or 1 μM formycin B plus 20 μg/mL of puromycin (NT2-KO). The culture was diluted (1:10, 1:25, 1:100) in a 24-well plate to grow individual clones. After 7–14 days, positive clones were grown. The positive clones were then picked from the 24-well plate and transferred to 10 mL of fresh preheated HOMEM media/10% FBS plus the appropriate selection agents. Gene deletion was confirmed using diagnostic PCR primers HDK1748/1749 and HDK1750/1751 for NT1-KO and NT2-KO, respectively (Supplemental Table S2). After confirmation, the process of NT1 allele deletion was repeated with a NT2-KO clone and, conversely, NT2-deletion with a NT1-KO clone was performed exactly as described above. Confirmation of the second-round knockout (generation of SUPKO) used the same primer pairs as used in the generation of the single gene-deletion strains.

### 4.6. qRT-PCR of Heterologously Expressed Genes

RNA was extracted using the NucleoSpin RNA kit (Macherey-Nagel, Düren, Germany) in accordance with the manufacturer’s instructions and quantified using a NanoDrop ND-1000 spectrophotometer; samples were stored at −80 °C until further use. qRT-PCR primers were designed using Primer Express 3.0 software (Supplemental Table S3). A total volume of 20 μL was obtained for each sample by diluting 4 μg of RNA in RNase-free water and cDNA was synthesized using the Precision nanoScript 2 Reverse Transcription kit (PrimerDesign Ltd., Eastleigh, UK) and stored at −20 °C. Primer efficiency was established using the method of Pfaffl [64]. The cDNA was amplified using the PrecisionPLUS OneStep RT-qPCR Master Mix kit (PrimerDesign Ltd., Eastleigh, UK) in a 7500 Real-Time PCR System coupled to a desktop computer (Thermo Fisher Scientific, Oxford, UK). Samples without reverse transcriptase (RT) or cDNA were used in the experiment as negative controls and the gene expression was normalized to GPI8 expression, which is a standard reference gene in *L. mexicana* [65]. Relative quantification was calculated using the delta delta ct method. Applied Biosystems 7500 Fast Real-Time PCR System Software (Thermo Fisher Scientific, Oxford, UK) was used for analysis of data. Each experiment was carried out with three independent determinations.

### 4.7. Alamar Blue Assay for Cell Survival and Drug Sensitivity

The assay is based on the reduction of the dye resazurin (Alamar blue) by live but not dead cells [66] and was performed with various strains of *Leishmania mexicana* promastigotes, exactly as described [67]. Briefly, doubling dilutions starting at 100 µM were set up over 2 rows of a 96-well plate and 2 × 10^5^ cells were added to each well, followed by incubation at 25 °C. After 72 h, 20 µL of 125 µg/mL resazurin solution was added and the plates incubated for a further 24 h. Fluorescence was determined in a FLUOstar OPTIMA plate reader (BMG Labtech, Ortenberg, Germany), reading the fluorescence intensity in arbitrary units (A.U.) at 544 nm wavelength for excitation and 620 nm wavelength for emission. The EC_50_ values and fluorescence data were determined and plotted to a sigmoid curve with variable slope, using the GraphPad Prism 8 software package. All EC_50_ values were determined on at least three independent occasions and are presented as average ± SEM. 

### 4.8. Transport Assays

Transport assays were performed exactly as described previously [2]. Briefly, 10^7^ cells in 100 μL of assay buffer (AB) were mixed with an equal volume of radiolabel at 2× concentration for a preset time. The incubation was terminated by the addition of 700 μL of “Stop solution”, being ice-cold 1 mM solution of unlabelled permeant, followed by immediate centrifugation through an oil layer in a microfuge at 13,000 rpm. This causes the cell pellet to reside under the oil layer and isolated from residual radiolabel in the assay buffer. The tubes were flash-frozen in liquid nitrogen and the bottom tip, containing the cell pellet, cut off and collected in a scintillation tube. Cells were solubilized by gentle shaking after addition of a 2% SDS solution, before the addition of scintillation fluid (Scintilogic U, Lablogic, Sheffield, UK); the tubes were left to stand in the dark overnight before counting in a 300SL Hidex Scintillation Counter (Lablogic). Linear and nonlinear regression analyses were carried out using GraphPad Prism versions 8.0 and higher. Statistical analyses and parameters of linear regression included the coefficient of determination r^2^, test for whether the slope is significantly different from zero (F-test), test for whether the line is significantly non-linear (runs test) and test whether two slopes are significantly different (F-test). Whether two data points of at least three replicates were significantly different from each other was determined by unpaired *t*-test. In this paper, individual experiments, always performed in triplicate or quadruplicate, are shown in the figures. However, all parameters, including *K*_m_, V_max_, *K*_i_, given are the average of at least three separate, independent determinations; errors given are standard errors, unless otherwise specified.

## Data Availability

Not applicable.

## Supplementary Materials

The following supporting information can be downloaded at: https://www.mdpi.com/article/10.3390/ijms23158139/s1.
