# Supplementary material for: Nucleoside Transport and Nucleobase Uptake Null Mutants in Leishmania mexicana for the Routine Expression and Characterization of Purine and Pyrimidine Transporters
Source: Int J Mol Sci. 2022 Jul 23;23(15):8139. doi: 10.3390/ijms23158139 (PMC9331716; doi:10.3390/ijms23158139)
Supplement: Supplementary file 1 [file ijms-23-08139-s001.zip › ijms-1808348-supplementary.pdf]

Supplemental Table S1. sgRNA sequences used in the CRISPR/cas9 knockout of *L. mexicana* nucleoside transporters

Allele targeted	Oligonucleotide	Sequence
NT1.1/NT1.2	sgRNA scaffold<br><br>(G00 primer; HDK1741)	5'AAAAGCACCGACTCGGTGCCACTTTTTCAAGTTGATAACGGACTAGCCTTATTTTAACTTGCTATTTCTAGCTCTAAAAC -3'
	UFP (HDK1742)	5'TTCTCCTTCACAGGTCAGCAACGAGCCCCAgtataatgcagacctgctgc-3'
	DRP (HDK1743)	5'TTTGCGTTGCCCACCGCTGGTCGTCCGCCCccaatttgagagacctgtgc-3'
	sgRNA-5'<br><br>(HDK1744)	5'gaaattaatacgactcactataggTTTGGATGCTGCCGCTGCGTgttttagagctagaaatagc-3'
	sgRNA-3'<br><br>(HDK1745))	5'gaaattaatacgactcactataggGCAGGCAACACATAAAGAGGgttttagagctagaaatagc-3'
NT2	sgRNA scaffold<br><br>G00 primer; HDK1741)	5'AAAGCACCGACTCGGTGCCACTTTTTCAAGTTGATAACGGACTAGCCTTATTTTAACTTGCTATTTCTAGCTCTAAAAC -3'
	UFP (HDK1755)	5'CCCCTCCACTGCAGCAGATTGGCCCGTCATactacccgatcctgatccag-3'
	DRP (HDK1754)	5'AACATGTCTCTTGCCATTACCCTGACCTACggttctggtagtggttccgg-3'
	sgRNA-5' (HDK1752)	5'gaaattaatacgactcactataggTACCCTGACTGATCAAAACTgttttagagctagaaatagc -3'
	sgRNA-3' (HDK1753)	5'gaaattaatacgactcactataggTAAATACCACGTTCCCACAGgttttagagctagaaatagc -3'

UFP, upstream forward primer; DRP, downstream reverse primer

Supplemental Table S2. PCR primers used in this project.

Primer name	Position	Restriction site	Sequence (5'UTR – 3'UTR)	Amplicon name
HDK1427	Forward	*Bgl*II	5’GATTAGATCTATGCGTTTCGGTCGCTT	*FurD*
HDK1428	Reverse	*Xho*I	5’GGTTCTCGAGTCAGTAAACAGCAAAAC	
HDK1537	Forward	*Bgl*II	5’GGCCGGAGATCTATGCCTTTTTTCAGTTCC	*TcrNB2*
HDK1538	Reverse	*Xho*I	5’CCGGCTCGAGCTATATGGCAAGCACAATA	
HDK1748	Forward	-	5’GCGAGAGCCGCAAGTGGTACGAAATG	NT1
HDK1749	Reverse	-	5’GCCGTTCGAGAAGCCGAAGATGACC	
HDK1750	Forward	-	5’GACTCCCGTGGTACCGCTTCGG	NT2
HDK1751	Reverse	-	5’CAGAGCGACACCGGGGATGATGC	
HDK340	Reverse	-	5’CGTGGAGCAGCTGAAGGACA	pNUS-HcN
MB39	Forward	-	5’ATGAAAAAGCCTGAACTCAC	*Hygromycin*
MB40	Reverse	-	5’ACTCTATTCCTTTGCCCTCG	
MB37	Forward	-	5’ATGATTGAACAAGATGGATTGC	*Neomycin*
MB38	Reverse	-	5’TCAGAAGAACTCGTCAAGAAG	
HDK282	Forward	-	5’CGAATTCATGGCCAAGCCTTTGTCT	*Blasticidin*
HDK283	Reverse	-	5’CGAATTCTTAGCCCTCCCACACATA	

Supplemental Table S3. qRT-PCR primers

Primer name	Position	Sequence (5'UTR to 3'UTR)	Gene
HDK1438	Forward	5’GTGGGCATTCACCCTTATCA	*FurD*
HDK1439	Reverse	5’CCTTACTGGGAAGCCATAACTC	
HDK568	Forward	5’GCTTCTCGCCGTCGTTGA	*Lmex-NT1*
HDK569	Reverse	5’ATGATCCAGCGCTGCTTGT	
HDK1565	Forward	5’ACTTGCCCGCCCACTACTA	*TcrNB2*
HDK1566	Reverse	5’CGTCAGGGCACCAGAAACA	
GPI8FL	Forward	5’GGCTGTCATTGTCTCCTCCT	*L. mexicana GPI8*
GPI8RL	Reverse	5’GTACATGGTAAGCGCATTGG	
